# Anthropometry and Physical Performance in 13-Year-Old Australian Talent-Identified Male and Female Athletes Compared to an Age-Matched General Population Cohort

**DOI:** 10.3390/children10020212

**Published:** 2023-01-25

**Authors:** Paul Larkin, Todd Carlon, Benjamin Sortino, Sam Greer, Tennille Cuttiford, Gyan Wijekulasuriya, Calvin Pane

**Affiliations:** 1 Institute for Health and Sport, Victoria University, Melbourne, VIC 3001, Australia; 2 Maribyrnong Sports Academy, Melbourne, VIC 3001, Australia

**Keywords:** physical fitness, performance data, speed, power, youth athletic development

## Abstract

Talent-identified male and female athletes are assumed to have greater speed and power than the general population at a given age. However, a comparison of the jump and sprint performance of an Australian cohort of male and female youth athletes from various sports to age-matched controls has not occurred. Therefore, the aim of this study was to compare anthropometric and physical performance markers between ~13-year-old talent-identified youth athletes and general population Australian youth. The anthropometry and physical performance in talent-identified youth athletes (n = 136, 83 males) and general population youth (n = 250, 135 males) were tested during the first month of the school year in an Australian high school within a specialized sports academy. Talent-identified females were taller (*p* < 0.001; d = 0.60), sprinted faster (20 m: *p* < 0.001; d = −1.16), and jumped higher (*p* < 0.001; d = 0.88) than general population youth females. Similarly, talent-identified males sprinted faster (20 m: *p* < 0.001; d = −0.78) and jumped higher (*p* < 0.001; d = 0.87) than general population youth males, but were not taller (*p* = 0.13; d = 0.21). Body mass was not different between groups for males (*p* = 0.310) or females (*p* = 0.723). Overall, youth, particularly females, who are trained in a variety of sports, exhibit greater speed and power during early adolescence compared to their age-matched peers, with anthropometric differences only occurring in females at 13 years of age. Whether talented athletes are selected because they exhibit these traits or whether speed and power are developed through sport participation requires further investigation.

## 1. Introduction

There are well-known health-related benefits associated with youth physical activity [1], including cardiorespiratory fitness [2], muscular fitness [3], bone health [4], and cardiometabolic health [5]. Furthermore, evidence suggests that physical activity reduces depressive symptoms in children and adolescents [6], and can have a positive effect on cognitive function and academic outcomes (e.g., school performance, memory and executive function) [7]. While researchers have examined the level and types of physical activity youth undertake to inform guidelines and recommendations [8], few have reported the physical profile of the general Australian youth population; in particular, speed (i.e., 20-m sprint) and power (i.e., vertical jump) measures. When anthropometric and physical performance profiles have been reported, it has generally been to highlight “at risk” groups and the potential impact of a lack of physical activity on health and well-being [9,10]. Therefore, it is important to develop benchmarks of anthropometry and physical performance which can be used to inform practitioners, such as medical professionals, physical education teachers, and youth sport coaches, when prescribing physical activity in the general population.

While there has been limited research investigating the general youth population, there has been extensive research profiling the anthropometric and physical performance measures of talented youth athletes [11,12]. By definition, talented youth athletes have been described as individuals with a special ability in a specific domain which places them in the top 10% of their peers, and have the potential for high-level performance in adulthood [13,14,15,16]. Profiling of anthropometric and physical performance measures has been used to inform performance benchmarks, talent identification, health and well-being, and return-to-play protocols for youth athletes [17,18]. For example, the ability to perform high-speed running actions [19] and jump higher [20] are important prerequisites for successful performance in many sports. To assess the sprint capacity of a youth athlete, maximum linear acceleration assessments have been used, with the most common being a maximal 20 metre (m) sprint [21,22,23]. Further, to measure lower limb power, countermovement jump (CMJ) performance has been shown to provide youth athletes with a competitive advantage [24]. A limitation of the current knowledge is that performance benchmarks and age-related differences are based on studies which focused on specific sports, and typically observed males [25]. Therefore, there is a need to explore these factors in both generally trained and female populations.

While studies have explored physical activity and performance levels in the general youth population and sport-specific contexts, there are still limited normative anthropometric and physical performance data for the Australian youth general population and talent-identified youth athletes. To the authors’ knowledge, there are no studies available that provide sex-specific anthropometric and physical fitness normative data for a trained and untrained Australian youth population (i.e., 13-year-olds). Therefore, the purpose of this cross-sectional study was to present sex-specific anthropometric (i.e., body height, body mass) and physical performance (i.e., CMJ; 5 m, 10 m, and 20 m sprint) reference values for Australian talent-identified and general population youths. A further aim of this study was to determine anthropometric and physical differences between these two populations. These findings will provide benchmark values and identify specific anthropometric and physical performance characteristics for the relevant population, and potentially inform physical activity recommendations.

## 2. Materials and Methods

### 2.1. Participants

A total of 386 individuals participated in this cross-sectional study. Participants were recruited from a single Australian public high school (Year 7 and 8). When considered by sex, there were 168 females (talent-identified: n = 53, age = 12.92 ± 0.60 years; general population: n = 115, age = 12.96 ± 0.65 years) and 218 males (talent-identified: n = 83, age = 13.15 ± 0.56 years; general population: n = 135, age = 13.04 ± 0.63 years) who participated in the study. 

Participants were included in the study if they met the following inclusion criteria: (1) attending the partner school, (2) being in Year 7 and Year 8 school grades, and (3) participating in physical education classes during the testing period (Term 1 2022, February–March). Within this group, 136 were talent-identified youth athletes who were selected for and attended the sports academy within the high school. Participants were selected for the academy based on coach expert opinion of their sporting ability at the age of selection (~12 years old). The aim of the academy is to provide a training environment which accelerates an individual’s progress within their nominated sports national talent pathway. The training environment of the academy included three 45 min formal strength and conditioning sessions per week with nationally accredited strength and conditioning coaches. Further, participants completed between two to three 90 min sport-specific training sessions. This school-based training environment (i.e., strength and conditioning; sport-specific training) complemented the external sport-specific national talent pathway training (i.e., club training; national training camps) the participants were undertaking. The other 250 individuals were regular high school students from the general population. These individuals were not a part of the academy and completed regular Australian high school curriculum, including Physical Education classes. 

The sole exclusion criterion was injury which prevented participation in the testing session. Therefore, any individual who had an injury which impeded their ability to perform the physical assessments on the testing day were excluded from the study. Ethical approval was gained from the lead institution’s research ethics board (HRE 21-064) which abides by the guidelines of the Declaration of Helsinki. Consent was obtained for all participants and parents of all participants prior to data collection.

### 2.2. Experimental Design

All participants completed a range of anthropometric (i.e., standing height; body mass) and physical performance measures (i.e., CMJ; 5 m, 10 m, and 20 m sprint), which were completed in one session and conducted by tertiary educated strength and conditioning coaches with over 5 years’ experience conducting this testing battery. The testing occurred in the same indoor venue with standardised environmental conditions for all participants. Prior to testing all electronic testing equipment was calibrated according to manufacturer standards. The height and body mass of participants were recorded, followed by a standardised 10-min warm up, led by a qualified strength and conditioning coach, which included familiarisation to the CMJ and 20 m sprint procedures. Participants then completed a CMJ followed by the 20 m sprint [26]. 

### 2.3. Measures

Height was measured using a wall-mounted stadiometer (Wedderburn, Sydney, Australia), with each participant removing socks and shoes. The stretch–stature method [27] was used to minimise technical error, with each participant instructed to inhale and hold a deep breath during the measurement. All measurements of height were taken at the end of the inhale with the headboard placed firmly on the vertex of the head and heels together and on the ground. Height was measured in centimetres (cm), with typical error of 1.0 cm [28].

Portable force plates (ForceDecks FD4000, VALD Performance, Brisbane, Australia) were used to measure body mass and CMJ [29]. Participants stood with their hands on their hips and feet shoulder width apart on each of the force places. After calibration, participants were instructed to step onto the plates and stand as still as possible to determine body mass. The participant was then instructed to perform a CMJ to a self-selected depth “as quickly and explosively as possible” on “GO” after a 3-2-1-GO countdown. Participants had to start and land on the force plates, keep their hands on their hips and have their knees extended during flight. If this did not occur, the jump was ruled as invalid and repeated. One familiarisation jump occurred before three jumps were completed in total, with the maximum jump height (cm) and body mass (kg) recorded for analysis.

A 20 m straight-line course on a basketball court was used to measure sprint performance. Electronic timing gates (Swift Performance Equipment, Lismore, Australia) were positioned at the start line as well as at 5 m, 10 m, and 20 m intervals. All participants started in a crouched position with the front foot touching the start line, back heel up, and no hands on the ground. Participants had to start from a stationary position and could not use a 3-point start position. Participants began whenever they were ready and were instructed to “run as fast as they could” until they reached cones placed at the 25 m mark. Three maximal sprints were undertaken with a 2 min recovery in between. The best split times for 5 m, 10 m, and 20 m were recorded in seconds, with typical error of measurement being 0.03 s [26].

### 2.4. Statistical Analysis

Descriptive statistics were used to summarise the data, with the mean, standard deviation (SD), minimum, and maximum for all measures (i.e., standing height; body mass; CMJ; 5 m, 10 m, and 20 m sprint performance across each group relative to sex). The assumptions of normality were assessed visually using a histogram for all outcome variables. As there were no deviations from a normal distribution, parametric statistical analyses were conducted. Due to the difference in population size between the talent-identified athletes and general population groups, a Welch’s *t*-test were used to determine between-group differences in physical performance measures. R Studio version 4.1.2 (RStudio, Boston, USA) was used for all data management and analysis, with the “rstatix” package providing the integrated *t*-test function (*t*_test(), with argument “var.equal = False” for Welch’s *t*-test). For the independent *t*-test, each of the physical performance measures were the dependent variable. These measures were: stand height (cm), body mass (kg), CMJ (cm), and 5 m, 10 m, and 20 m sprint (seconds). For each analysis, the independent variable was the participant group (i.e., talent-identified athletes; general population). Point estimates for mean difference in populations, along with 95% confidence intervals, were calculated. Results were considered statistically significant for *p* < 0.05. Effect sizes were calculated by Cohen’s d [30] and the magnitude was described by Sawilowsky’s [31] rules of thumb (very small, d = 0.01; small, d = 0.20; medium, d = 0.50; large, d = 0.80; very large, d = 1.20; huge, d = 2.00).

## 3. Results

Table 1 and Table 2 present the mean and standard deviation values of the physical fitness tests for the talent-identified and general population relative to sex. In particular, female talent-identified athletes were taller (*p* < 0.001; d = 0.60), jumped higher (CMJ; *p* < 0.001; d = 0.88) and sprinted faster (5 m *p* < 0.001; d = −0.69; 10 m *p* < 0.001; d = −1.05; 20 m *p* < 0.001; d = −1.16) than their general population counterparts. There was no significant difference in body mass between the female groups (*p* = 0.723; d = −0.06).

In relation to males, talent-identified athletes were found to jump higher (CMJ; *p* < 0.001; d = 0.87) and sprint faster (10 m; *p* < 0.001; d = −0.62; 20 m; *p* < 0.001; d = −0.78) than their general population counterparts. There were no significant statistical differences for stand height (*p* = 0.13; d = 0.21), 5 m sprint (*p* = 0.07; d = −0.25), and body mass (*p* = 0.31; d = −0.13) between the groups.

## 4. Discussion

The aim of this study was to present sex-specific anthropometry and physical performance normative data for 13 year olds and to determine the difference between talent-identified and general population youth in anthropometry, speed, and power. Male and female youth talent-identified athletes exhibited greater speed over 10 m and 20 m and lower body power compared to age-matched general population youth. Female youth talent-identified athletes were taller than the general population, and body mass was not different between groups for both sexes. Therefore, speed and power are physical characteristics which discriminate talent-identified athletes from the general population in a heterogenous sample of youth. Anthropometric characteristics carry greater importance in differentiating talented females than talented males at ~13 years of age.

Our data suggest that speed and power are discriminating factors between talent-identified male and female youth athletes and the general population. There are limited studies which compare the speed and power of trained and untrained youth male and female populations. Estonian girls (10 to 17 years old) who participated in track and field were found to have greater 30 m sprint speed and CMJ jump height for all chronological age groups than a recreationally active control group [32]. Russian girls trained in judo and volleyball aged 12 to 14 years were faster and had a longer standing broad jump compared with untrained youth [33], and male swimmers (~14 years old) had greater upper body strength compared to age-matched controls [34]. The principle of training specificity can explain the differences of previous studies, as youth competing in sports which require speed [32], lower body power [32,33], and upper body strength [34] had greater performance of these physical characteristics compared to untrained youth. However, the results of the current study show that differences in speed and lower body power exist between the general population and a heterogenous sample of youth talent-identified athletes. Further work is required to determine whether improvements in speed and power are caused by participation in sport alone and the extent that these potential adaptations may have on the health of athletes and youth in general. 

Previous research has shown that despite no resistance training, male 14-year-old recreational and talent-identified soccer players have greater strength during high- and low-velocity concentric contractions of lower limb muscles compared to untrained controls [35]. For youth aged ~13 years, predominantly neuromuscular adaptations increase speed and power [36]. Specifically, greater motor unit activation, neuromuscular coordination and neural drive is purported to increase strength and power in youth aged between 12 and 14 years [37]. This is because increases in muscle size and mass and strength gains associated with these morphological changes typically occur after the period of peak growth (peak height velocity) in youth [37,38]. Resistance training is a modality which increases strength, speed, and lower body power in youth athletes [39,40]. The talent-identified population within this study participated in resistance training based program within the sports academy; therefore, training adaptation to this stimulus may explain the differences in speed and lower body power. However, testing occurred in Term 1 of the school calendar, with participants also having had limited structured resistance training during the 12 months prior to testing due to COVID-19 lockdown restrictions, and had limited structured resistance training for the 6 weeks prior to testing. Since resistance training history was not collected within each cohort, there is no way of knowing the extent that residual strength, speed, and power gains from previous training explained the differences in speed and power in the cohorts of talent-identified and general population youth studied. A previous study of ~16-year-old male youth athletes have suggested that resistance training age predicts lower body power but sport training age predicts strength and athletic qualities like change of direction ability [41]. Further research is required to determine the extent to which sport exposure and/or resistance training exposure affects speed and power, given that these physical characteristics discriminate between talent-identified youth and the general population. 

The magnitude of difference between the anthropometric and physical characteristics of talent-identified and untrained male and female 13-year-olds was sex-specific. Talent-identified females were taller than their sex-matched general population counterparts, whilst height was not different between male cohorts. Furthermore, there were consistent differences and effect sizes between male and female cohorts for the CMJ, whilst the difference in sprinting was greater in magnitude between trained and untrained females compared to males. This is the first study, to the authors’ knowledge, to observe the differences in anthropometric and physical characteristics between trained and untrained male and female youth. Taller talent-identified females compared to the general population may be due to the talent identification practices in certain sports. For example, there is a general understanding that height is a consideration when identifying and selecting athletes for sports such as netball [42], basketball [43], and volleyball [44]. Therefore, as there were a large number of female athletes from these sports, the identified difference may be due to an over-representation of taller female athletes selected for these sports, rather than a consistency across the female youth athlete population. 

In contrast, while male talent-identified athletes also participate in sports (e.g., basketball, volleyball, Australian Rules Football) where height is an advantage, there was no difference in height between talent-identified and general population young males. Whilst the heterogeneity of the sports within the sports academy improves the generalisability of the results to the wider cohort of “talent-identified youth” and reduces the likelihood of biased athletic profiling, the physical performance and anthropometric variables in the athlete cohorts may be influenced by the distribution of athletes between different sports. Furthermore, females experience peak height velocity (PHV) at an earlier age than males [36], and talent-identified females may experience this at an even younger age; therefore, they may be within or even past this period by the age of 13, which may also explain the greater difference in height. This would also explain the greater magnitude of speed differential between general population and talent-identified females compared to similar cohorts of males, who would most likely be pre-PHV, and the greater speed and power of talented youth compared to the general population. A major limitation of the study design is that maturation status was not assessed in either population; therefore, it is unknown whether biological maturity affected speed and power measures. Future studies comparing talent-identified and/or trained youth should collect and report biological maturation using common field-based measures [45] to help elucidate whether maturation or training status is the predominant predictor of speed and power in 13-year-olds, as well as other cohorts of age-matched talent-identified and untrained youth [46]. 

The results also highlighted a similar increase in CMJ performance between groups in the male and female populations; however, the reasoning for this large effect is more difficult to explain. Males and females have similar lower body power until they are ~13 years old, when the rate of increase in lower body power changes in a sex-dependent manner [47]. Accordingly, males of matched-talent groups have greater CMJ scores compared to females. The similarity in effect may be a statistical anomaly, as the cross-sectional design of this study limits its generalisability. Using a longitudinal study design where male and female general population and talent-identified youth are monitored for numerous years may also overcome this limitation and help determine whether a global difference in lower body power exists between trained and untrained youth, or whether these effects change over time. 

While a strength of the study is the collection of a large heterogeneous sample, interpretation of the results should, however, be considered with respect to methodological limitations. Specifically, as the current study is cross-sectional in nature, data were only based on current performance. Future studies should consider using a more longitudinal design to not only confirm current results, but also assess for potential anthropometrical and physical performance changes over several time periods. Additionally, the study does not consider the participants’ maturation status; therefore, the data only provide a generalized view of the populations. Future studies should consider incorporating measures of maturation, such as peak height velocity [48] and the impact of this on the individual measures. Additionally, replication of this study in cohorts of youth of different ages, in different training environments, and of different ethnicities, is warranted. More studies assessing the effect of resistance training on the speed and power of talent-identified and general population youth would help determine the efficacy of resistance training modalities in bridging the gap in physical fitness between untrained and trained young people.

## 5. Conclusions

Overall, male and female talent-identified youth (~13 years old) athletes are faster and have greater lower body power than age-matched general population youth. Female talent-identified youth are taller, and there is no difference in body mass between talented and non-talent-identified youth. The extent that training and competing in sport and/or biological maturation modulate these population differences is unknown. Physical performance and anthropometric normative data about talent-identified and general population male and female youth are presented for strength and conditioning coaches, physiotherapists, and other allied health professionals to use to guide their interpretation of the results of similar assessments in similar cohorts. Future research should replicate this study design with different age groups, or utilise a longitudinal design to develop a greater understanding of the relationship between training, maturation, and physical performance of talent-identified athletes and the general population.

## 6. Practical Applications

- Speed and power discriminate male and female talent-identified youth athletes (~13 years old) from the general population.
- Youth strength and conditioning coaches, allied health professionals, and physical educators who facilitate the development of youth athletes now have physical performance benchmarks which can be used to guide training prescription.
- The development of speed and power prior to the age of 13 years may be beneficial to improve athletic and sports performance for males and females.

## Figures and Tables

**Table 1:** Descriptive statistics with group difference for female talent-identified athletes and the general population counterparts for the physical performance measures.

	Talent-Identified Athletes (n = 53)			General Population (n = 115)							
	Mean (SD)	Maximum	Minimum	Mean (SD)	Maximum	Minimum	Mean Difference	Lower 95% CI	Upper 95% CI	*p*-value	Effect Size
Stand Height (cm)	160.93 (7.40)	142.7	180.19	156.91 (5.89)	144.4	170.7	4.02	1.72	6.32	<0.001 *	Medium
Body Mass (kg)	51.27 (8.71)	32.35	74.99	51.84 (11.59)	31.50	105.70	−0.57	−3.76	2.62	0.723	Very Small
CMJ (cm)	24.86 (4.02)	17.10	32.60	20.70 (4.96)	6.50	32.10	3.98	2.55	5.40	<0.001 *	Large
5 m Sprint (s)	1.26 (0.07)	1.12	1.44	1.32 (0.11)	1.12	1.71	−0.07	−0.10	−0.04	<0.001 *	Medium
10 m Sprint (s)	2.08 (0.11)	1.90	2.35	2.24 (0.18)	1.95	2.92	−0.15	−0.20	−0.11	<0.001 *	Large
20 m Sprint (s)	3.61 (0.19)	3.29	4.09	3.94 (0.36)	3.16	5.38	−0.34	−0.42	−0.25	<0.001 *	Large

* denotes a significant (*p* < 0.05) difference between groups, SD: standard deviation.

**Table 2:** Descriptive statistics with group difference for male talent-identified athletes and the general population counterparts for the physical performance measures.

	Talent-Identified Athletes (n = 83)			General Population (n = 135)							
	Mean (SD)	Maximum	Minimum	Mean (SD)	Maximum	Minimum	Mean Difference	Lower 95% CI	Upper 95% CI	*p*-value	Effect Size
Stand Height (cm)	163.1 (10.66)	145.4	190.44	160.93 (9.85)	136.6	191.6	−1.86	−0.68	5.03	0.134	Small
Body Mass (kg)	52.43 (10.46)	32.19	81.84	54.29 (16.67)	26.10	121.20	2.18	−5.49	1.76	0.311	Very Small
CMJ (cm)	29.32 (6.26)	16.10	43.30	24.08 (5.77)	8.70	37.10	5.24	3.57	6.92	<0.001 *	Large
5 m Sprint (s)	1.22 (0.09)	1.03	1.48	1.25 (0.10)	1.02	1.71	−0.02	−0.05	0.00	0.069	Small
10 m Sprint (s)	2.01 (0.13)	1.71	2.37	2.11 (0.17)	1.76	2.92	−0.10	−0.14	−0.05	<0.001 *	Medium
20 m Sprint (s)	3.49 (0.24)	2.94	4.08	3.72 (0.36)	3.08	5.20	−0.24	−0.32	−0.16	<0.001 *	Medium

* denotes a significant (*p* < 0.05) difference between groups, SD: standard deviation.

## Data Availability

The data presented here are available on request from the corresponding author. The data are not publicly available due to ethical and privacy matters.
